# 2749. Impact of rapid carbapenemase identification on management of patients with carbapenem-resistant Enterobacterales infections

**DOI:** 10.1093/ofid/ofad500.2360

**Published:** 2023-11-27

**Authors:** Elysia Burke, Lauren Dutcher, Keith W Hamilton, Kathleen Degnan, Laurel Glaser, Christina Maguire, Stephen Saw, Katherine Mersinger, Sonal Patel, Vasilios Athans, Shawn Binkley

**Affiliations:** UPMC Harrisburg, Enola, Pennsylvania; University of Pennsylvania Perelman School of Medicine, Philadelphia, Pennsylvania; University of Pennsylvania Perelman School of Medicine, Philadelphia, Pennsylvania; University of Pennsylvania Perelman School of Medicine, Philadelphia, Pennsylvania; University of Pennsylvania, Philadelphia, Pennsylvania; Penn Presbyterian Medical Center, Philadelphia, Pennsylvania; Hospital of the University of Pennsylvania, Philadelphia, Pennsylvania; Hospital of the University of Pennsylvania, Philadelphia, Pennsylvania; Hospital of the University of Pennsylvania, Philadelphia, Pennsylvania; Hospital of the University of Pennsylvania, Philadelphia, Pennsylvania; Hospital of the University of Pennsylvania, Philadelphia, Pennsylvania

## Abstract

**Background:**

Carbapenem-resistant Enterobacterales (CRE) infections can lead to increased length of stay and mortality. Identification of the mechanism of resistance is vital to assist in antibiotic selection. In October 2021, our institution’s microbiology laboratory switched from the CARBA NP assay, which detects carbapenemase production, to the CARBA 5 assay, which detects and differentiates types of carbapenemases. The primary aim was to characterize the pattern of carbapenemases within our institution. The secondary aim was to identify whether the transition to the CARBA 5 assay impacted antibiotic management.

**Methods:**

This was a quasi-experimental, pre-post study design, including adults with at least one isolate of carbapenemase-producing Enterobacterales (CPE) from October 2020 – October 2022. The CARBA 5 testing protocol began on October 27^th^, 2021. The pretest-posttest groups were assigned before and after this date, respectively. For patients with multiple CPE isolates, only the first isolate per patient was included. Patients who were deemed to be colonized (not treated) were not included in outcome analysis. Descriptive statistics were used to characterize demographics, distribution of carbapenemase type, and colonization. Time-to-appropriate therapy was defined as time to administration of the first antibiotic with known susceptibility. All time-based outcomes were measured from the time the culture resulted as carbapenem-resistant in the medical record.

**Results:**

The pretest group had 49 patients, of which 22 were treated. The posttest group had 37 patients, of which 17 were treated. Distribution of carbapenemases and pathogens are listed in Table 1 and Table 2. The median time-to-appropriate therapy was 5.3 hours (IQR 3.2-23.9) in the pretest group versus 5.0 hours (IQR 3.3-10.7) in the posttest group. Of the patients treated, 6 (26.1%) patients experienced 30-day mortality in the pretest group versus 4 (22.2%) in the posttest group. Median time-to-discharge in the pretest group was 14.7 (IQR 7.6-19.2) days versus 9.6 days (IQR 5.7-15.3) in the posttest group.

**Figure:**
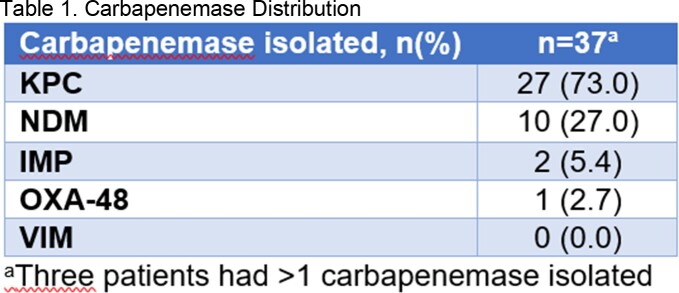

**Figure:**
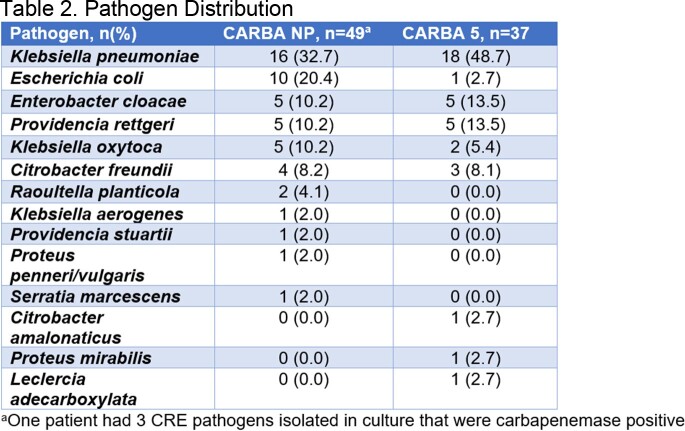

**Conclusion:**

The most common carbapenemase was KPC; the most common pathogen was *Klebsiella pneumoniae.* Time-to-appropriate therapy and time-to-discharge during the CARBA 5 test period were numerically lower.

**Disclosures:**

**Kathleen Degnan, MD**, Gilead: Grant/Research Support **Christina Maguire, PharmD**, Viiv: Advisor/Consultant

